# Removal of As(III) from Biological Fluids: Mono- versus Dithiolic Ligands

**DOI:** 10.1021/acs.chemrestox.9b00506

**Published:** 2020-03-17

**Authors:** Donatella Chillè, Giuseppe Cassone, Fausta Giacobello, Ottavia Giuffrè, Viviana Mollica Nardo, Rosina C. Ponterio, Franz Saija, Jiri Sponer, Sebastiano Trusso, Claudia Foti

**Affiliations:** † Dipartimento di Scienze Chimiche, Biologiche, Farmaceutiche e Ambientali, Università di Messina, Viale F. Stagno d’Alcontres 31, 98166 Messina, Italy; ‡ CNR-IPCF, Viale Ferdinando Stagno d’Alcontres 37, 98158 Messina, Italy; § Dipartimento di Scienze Matematiche e Informatiche, Scienze Fisiche e Scienze della Terra, Università di Messina, Viale F. Stagno d’Alcontres 31, 98166 Messina, Italy; ∥ Institute of Biophysics of the Czech Academy of Sciences, Kràlovopolskà 135, 61265 Brno, Czech Republic

## Abstract

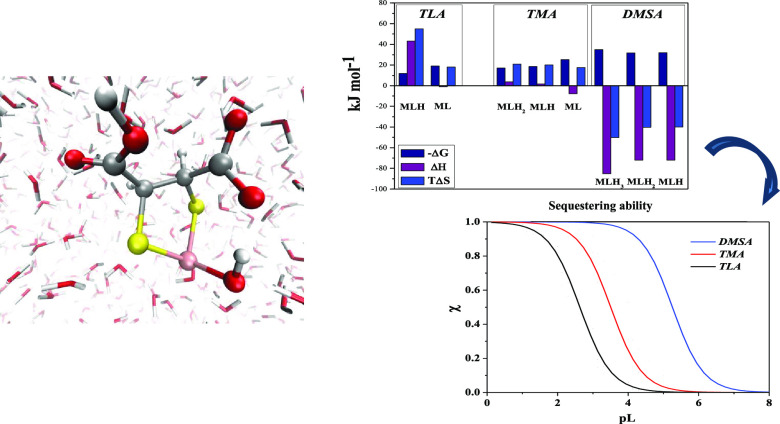

Arsenic is one of the inorganic pollutants typically found in natural waters, and its toxic effects on the human body are currently of great concern. For this reason, the search for detoxifying agents that can be used in a so-called “chelation therapy” is of primary importance. However, to the aim of finding the thermodynamic behavior of efficient chelating agents, extensive speciation studies, capable of reproducing physiological conditions in terms of pH, temperature, and ionic strength, are in order. Here, we report on the acid–base properties of *meso*-2,3-dimercaptosuccinic acid (DMSA) at different temperatures (i.e., *T* = 288.15, 298.15, 310.15, and 318.15 K). In particular, its capability to interact with As(III) has been investigated by experimentally evaluating some crucial thermodynamic parameters (Δ*H* and *T*Δ*S*), stability constants, and its speciation model. Additionally, in order to gather information on the microscopic coordination modalities of As(III) with the functional groups of DMSA and, at the same time, to better interpret the experimental results, a series of state-of-the-art *ab initio* molecular dynamics simulations have been performed. For the sake of completeness, the sequestering capabilities of DMSA—a simple dithiol ligand—toward As(III) are directly compared with those recently emerged from similar analyses reported on monothiol ligands.

## Introduction

Arsenic is a ubiquitous element easily found in the environment deriving from both natural and anthropogenic sources. It is a component of the Earth’s crust, it is also present in minerals and soils, hence potentially reaching water and air through wind-blown dust and runoff. In addition, its usage in industrial processes, mining activities, pesticides, and fertilizers contributes to the global contamination as a consequence of leaching procedures. The prevalent forms that can be found in the environment are inorganic, especially arsenate (As(V)) and arsenite (As(III)), exhibiting different toxicities on the human organism as well as diverse species distributions depending on pH.^1,2^ In 1963, the World Health Organization fixed a recommended value for arsenic in drinking water equal to 50 μg L^–1^, which was successively further reduced to 10 μg L^–1^, as a consequence of the suspicion of carcinogenicity.^3^ Arsenic is now recognized as one of the most dangerous inorganic pollutants; therefore, the development of removal methods is necessary.

Historically, the use of chelating agents for decreasing metal or metalloid toxicity dates back to about 100 years ago in order to alleviate the toxic effects of arsenic compounds used for syphilis treatment.^4−6^ In general, removal of metals takes place through the formation of non-toxic complexes which must be water-soluble, stable, and easily excreted in the urine. In fact, the ideal chelating agent should have high solubility in water, low toxicity, and ability to penetrate cell membranes; furthermore, it should be resistant to biotransformation and have a high affinity for toxic metals at the pH of body fluids.^7,8^ Unfortunately, most of the available chelating agents have limited ability to remove metals from the brain tissue since they are not capable to cross the blood–brain barrier.^9,10^

*meso*-2,3-Dimercaptosuccinic acid (DMSA) is a sulfhydryl-containing compound potentially useful in chelation therapy to remove heavy metals from body fluids.^11^ In particular, it has been approved for the removal of lead by the U.S. Food and Drug Administration.^12^ It is able indeed to form stable and water-soluble complexes with lead which are easily excreted by urine, reducing hence the lead content in the brain. Besides, DMSA chelates also other heavy metals such as Cd^2+^ and Hg^2+^, but, due to the presence of highly charged carboxyl groups in its structure, it cannot enter a cell membrane. In this context, by means of experimental thermodynamic techniques and state-of-the-art *ab initio* molecular dynamics simulations, the possibility of employing DMSA as chelating agent for As(III) because of the pronounced affinity of the latter toward the −SH groups is here investigated. It is worth mentioning that the nature and the stability of the formed complexes are the necessary requisites, though not sufficient, such that the metal ion can be completely transformed into the chelated species to be excreted.^8^ In this Article, the aim is to evaluate the use of DMSA as sequestering agent for As(III) from the thermodynamic point of view and to understand the mechanism of interactions. Both these aspects represent the starting point for further developments. Therefore, the knowledge of the chemical binding forms, i.e., speciation, in As(III)-DMSA system can be of great importance to understand not only its biokinetics but also its relevance in risk assessment and in designing chelation therapy in the case of overexposure. In order to evaluate the effect of the presence of thiol groups on the binding ability, results are reported in direct comparison to those recently published for some monothiol ligands, such as 2-mercaptopropionic acid (or thiolactic acid, TLA) and 2-mercaptosuccinic acid (or thiomalic acid, TMA).^13^ The sequestering ability of thiolic ligands toward As(III) is evaluated by the pL_0.5_ empirical parameter which represents the ligand concentration required to sequester 50% of the metal cation present in traces.

## Experimental Section

### Reagents

Arsenic(III) solutions were prepared by weighing the sodium (meta)arsenite salt (Sigma-Aldrich, ≥90%). *meso*-2,3-Dimercaptosuccinic acid solutions were prepared by weighing the corresponding Sigma-Aldrich product used without further purification. Purity was checked potentiometrically and was always greater than 98%. Standard solutions of HCl and NaOH were prepared from concentrated Fluka ampules and titrated with sodium carbonate and potassium biphthalate, respectively, previously dried in an oven at 383.15 K for at least 1 h. Sodium hydroxide solutions were therefore stored in dark bottles and preserved by CO_2_ by means of soda lime traps. Sodium chloride solutions were prepared by weighing the corresponding Fluka salt, pre-dried in an oven at 383.15 K. Grade A glassware and bi-distilled water were used for the preparation of all the solutions.

### Equipment

Potentiometric measurements were carried out by an automatic system consisting of 809 Metrohm Titrando equipped with combination glass electrode Ross type 8102, from Thermo-Orion. The apparatus was controlled by Metrohm TiAMO 1.2 software able to track the emf stability, titrant delivery, and data acquisition. Estimated precision is ±0.15 mV for the emf and ±0.003 mL for titrant volume readings. The temperature was kept constant at *T* = 288.15, 298.15, 310.15, and 318.15 ± 0.1 K by using thermostated glass jacket cells under magnetic stirring to ensure homogeneity of the systems.

The spectrophotometric measurements on aqueous solutions were recorded using a Varian Cary 50 UV–vis spectrophotometer equipped with an optic fiber probe having a fixed 1 cm path length. The spectrophotometer was connected to a PC for the acquisition of the experimental data (absorbance vs wavelength) by Varian Cary WinUV (version 3.00) software. Simultaneously, pH vs volume of titrant (mL) data were recorded by using a combined glass electrode (Ross type 8102, from Thermo/Orion) connected to a Metrohm 713 potentiometer. The titrant was delivered in the measurement cell by means of a 665 Metrohm automatic buret and, also in this case, the solutions were vigorously stirred in order to keep homogeneous the systems during all the titration processes. The combined glass electrode was standardized before each experiment in terms of pH = −log [H^+^]. Preliminary absorbing spectra were previously recorded to know the wavelength interval where the ligand absorbs; the selected wavelength range was from λ = 200 to 350 nm.

### Procedure and Calculations

Both potentiometric and spectrophotometric measurements were carried out as titrations. In the study of the H^+^-DMSA system, 25 mL of solutions containing DMSA, HCl (necessary to fully protonate the ligand), and NaCl (necessary in order to fix the ionic strength) were titrated by means of standard NaOH in a wide range of pH (2 ≤ pH ≤ 10.5). In the study of the As(III)-DMSA system, 25 mL of the solutions containing As(III), DMSA, HCl, and NaCl were titrated via standard NaOH in the same pH range (2 ≤ pH ≤ 10.5). Experimental details are reported in Table 1. When the potentiometric technique was used, independent titrations of HCl with standard NaOH were performed in order to determine the standard electrode potential, *E*^0^, and p*K*_w_ values in the same experimental conditions of ionic strength and temperature. All titrations were performed on bubbling purified pre-saturated N_2_ through the solution to exclude O_2_ and CO_2_ inside.

**Table 1:** Conditions for the Experimental Study of H^+^-DMSA and As(III)-DMSA Systems

technique	*C*_M_^a^	*C*_L_^a^	*C*_M_/*C*_L_	*C*_HCl_^a^	*C*_NaCl_^b^	*T*/K
potentiometry	–	1–3	–	5–10	0.15	288.15, 298.15, 310.15, 318.15
	1–2	1–3	0.5–2	5–10	0.15	288.15, 298.15, 310.15, 318.15

spectrophotometry	–	0.05–0.15	–	1–3	0.15	298.15
	0.05–0.15	0.05–0.15	0.5–2	1–3	0.15	298.15

a In mmol L^–1^.

b In mol L^–1^.

The BSTAC software^14^ was used to refine all the parameters (protonation and formation constants, analytical concentration of reagents, formal electrode potential, acid junction potential, and ionic product of water) of the potentiometric titrations. Finally, the HYSPEC computer program^15^ was used to analyze the UV–vis spectra and to calculate the stability constants and the molar absorbance of each formed species.

## Computational Section

A numerical sample composed of one DMSA, along with 100 water molecules and one arsenic As(III) atom (i.e., 317 atoms in total), has been arranged in a cubic simulation box with edge equal to 14.88 Å – conferring hence to the box a density of 1.05 g/cm^3^ – while, as usual, periodic boundary conditions were applied. Albeit the simulated sample reproduces a concentration which is order of magnitudes higher than those experimentally encountered, going beyond such a concentration is computationally unfeasible via *ab initio* molecular dynamics. Moreover, since we are uniquely interested in the atomistic chelation modalities, performing those simulations at higher nominal concentrations—provided that an adequate level of solvation of the chelated complex is provided—cannot represent a concrete limitation of the computational approach here adopted.

The starting molecular configuration was prepared by means of classical molecular dynamics by employing standard force-fields and by running the respective simulation for 5 ns. The resulting configuration has been then equilibrated via accurate first-principles molecular dynamics for 5 ps, after which an accumulation run 100 ps long was initiated. Different arsenic–DMSA starting distances, topologies, and initial velocities (taken from a Maxwell–Boltzmann distribution) have been tested in order to check the effects of the starting structure/forces on the chelation properties and dynamics.

The same protocol has been executed for samples containing, on the one hand, one thiolactic acid (TLA) species, one arsenic atom, and 70 water molecules (i.e., 223 atoms), and, on the other, thiomalic acid (TMA) solvated by 70 water molecules and one arsenic atom (i.e., 227 atoms), as reported in ref (13). While in the former numerical sample an edge of the cubic box equal to 13.39 Å has been chosen, in the latter a cubic side of 13.51 Å has been adopted. In all cases, since As(III) carries a charge of +3, a compensating *jellium* background has been added in order to avoid the divergences due to the infinite replica of the (charged) simulation boxes.^16^

We used the software package CP2K,^17^ based on the Born–Oppenheimer approach, to perform *ab initio* molecular dynamics simulations of the above-mentioned samples. Electronic wave functions of each atomic species have been expanded on a mixed basis set composed of extended plane waves and local Double Zeta Valence plus Polarization (DZVP) basis sets. As for exchange and correlation (XC) effects, we adopted the gradient-corrected Becke–Lee–Yang–Parr (BLYP)^18,19^ functional in conjunction with D3(BJ) Grimme’s dispersion corrections,^20,21^ whereas Goedecker–Teter–Hutter pseudopotentials^22^ have been chosen to mimic the core electronic interaction. A cutoff energy for the wave functions representation of 40 Ry and a cutoff of 400 Ry for the charge density have been employed whereas a time step of 0.5 fs, typical for Born–Oppenheimer molecular dynamics, has been chosen for each of the trajectories. All the *ab initio* molecular dynamics simulations have been carried out at the average temperature of 300 K controlled by means of the Canonical Sampling through Velocity Rescaling method.^23^ In this way, samples were simulated in an isothermal–isochoric (NVT) ensemble, and the dynamics of the nuclei was classically propagated using the Verlet algorithm. Hydration properties of simple ions and of relatively complex compounds have been previously extensively tested by some of our group through those first-principles molecular dynamics simulation techniques (see, e.g., refs (1, 13, 24−27)).

## Results and Discussion

### Acid–Base Properties of DMSA

**Figure 1:**
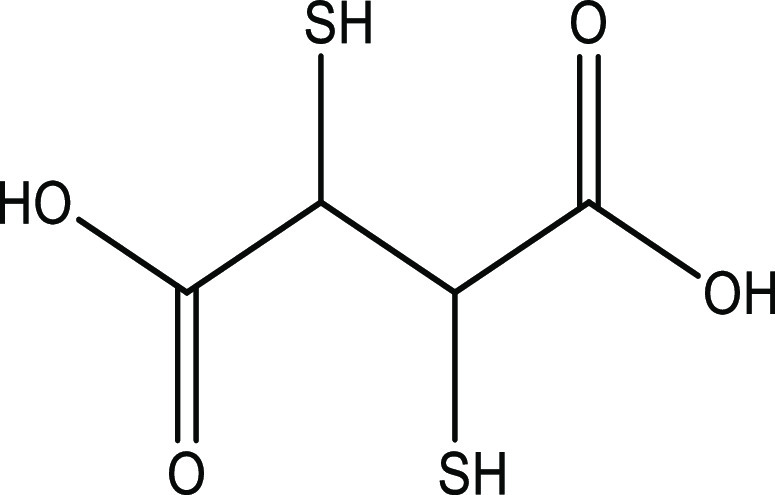
*meso*-2,3-Dimercaptosuccinic acid (DMSA).

The binding ability of DMSA (Figure 1) has been studied in NaCl at *I* = 0.15 mol L^–1^ for different temperatures in order to evaluate the enthalpy values and, therefore, to obtain a complete picture of the thermodynamic parameters. The first step was the definition of the acid–base properties of the ligand at the same ionic strength and temperature conditions of metal–ligand system.

DMSA contains four protonable groups, two carboxylic and two thiolic, each of which exhibiting very similar properties due to the intrinsic molecular symmetry. In order to define all of them, potentiometric titrations were carried out on solutions containing variable ligand concentrations, in the range 1 ≤ *C*_L_ ≤ 3 mmol L^–1^ and at different temperatures (288.15 ≤ *T* ≤ 318.15 K). Results are reported in Table 2. Clearly, to the aim of associating the protonation constants to each group, stepwise equilibria have to be considered as in reaction 1:
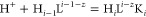
1In this way, the relative protonation constants are log *K*_*i*_ = 11.01, 9.31, 3.55, and 2.52, for *i* = 1, 2, 3, and 4, respectively, at *T* = 298.15 K. Whereas the first two values refer to the protonation of thiolic groups, K_3_ and K_4_ refer to the carboxylic ones.

**Table 2:** Experimental Protonation Constant Values of DMSA in NaCl at *I* = 0.15 mol L^–1^

	log β^a^			
reaction	288.15 K	298.15 K	310.15 K	318.15 K
H^+^ + L^4–^ = HL^3–^	10.96 ± 0.02	11.01 ± 0.04	11.27 ± 0.02	11.32 ± 0.03
2H^+^ + L^4–^ = H_2_L^2–^	20.15 ± 0.03	20.32 ± 0.03	20.64 ± 0.02	20.82 ± 0.03
3H^+^ + L^4–^ = H_3_L^–^	23.51 ± 0.05	23.87 ± 0.02	24.35 ± 0.02	24.61 ± 0.02
4H^+^ + L^4–^ = H_4_L^0^	26.14 ± 0.04	26.39 ± 0.03	26.85 ± 0.02	27.09 ± 0.02

a ±standard deviation.

**Figure 2:**
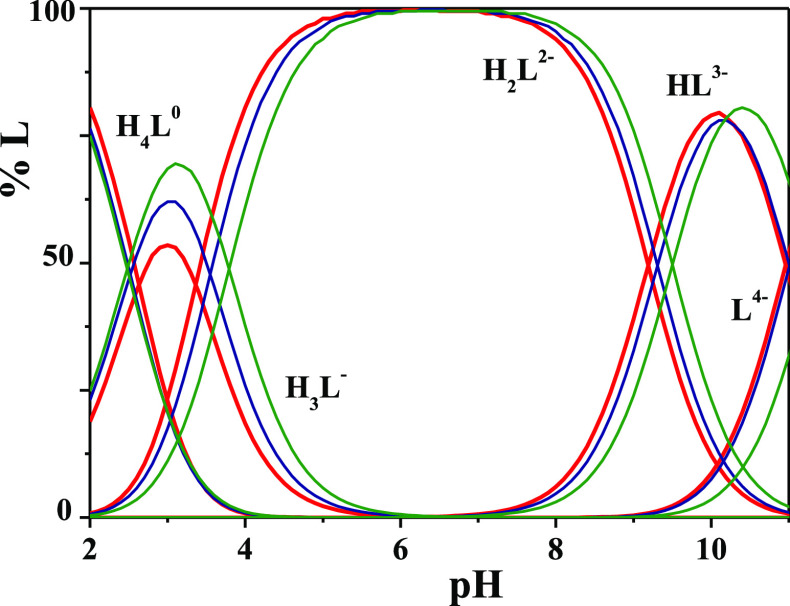
Distribution diagram vs pH of H^+^-DMSA species. Experimental conditions: *C*_L_ = 2 mmol L^–1^, *I* = 0.15 mol L^–1^ (NaCl), and *T* = 288.15 (red lines), 298.15 (blue lines), and 318.15 K (green lines).

As can be observed from the distribution diagram of the H^+^-DMSA species reported in Figure 2, tetra- and tri-protonated species prevail for pH < 4. In a wide pH range (4 < pH < 9), the ligand is present as H_2_L, and only for pH > 8 deprotonation of thiolic groups is triggered.

The effect of the temperature on the percent fraction of species formation is negligible for H_2_L (which represents the main species at physiological conditions, pH = 7.4) while an increment from 50% to 70% as regards the H_3_L formation is recorded at pH = 3 when the temperature is increased from 288.15 to 318.15 K, as shown in Figure 2.

### As(III)-DMSA System

In order to investigate the interaction between As(III) and DMSA, potentiometric and spectrophotometric titrations were performed by using different metal/ligand ratios at *I* = 0.15 mol L^–1^ and at different temperatures (i.e., *T* = 288.15, 298.15, 310.15, and 318.15 K). After several trials, elaboration of data allowed us to define the best speciation model, taking into account the best correspondence between the experimental and the calculated curves, the simplicity, the probability, and the percentages of species formation as well as the stability constants of the complex species.^28,29^ For the As(III)-DMSA system, only mono-coordinated species, differently protonated, were determined, and their stability constants are listed in Table 3.

**Table 3:** Experimental Formation Constant Values of As(III)-DMSA Species in NaCl at *I* = 0.15 mol L^–^^1^

	log β^a^			
species	288.15 K	298.15 K	310.15 K	318.15 K
MLH_3_	30.02 ± 0.05	29.97 ± 0.08	29.85 ± 0.07	29.76 ± 0.07
MLH_2_	26.10 ± 0.06	25.87 ± 0.09	25.66 ± 0.08	25.45 ± 0.07
MLH	16.85 ± 0.05	16.59 ± 0.09	16.32 ± 0.08	16.07 ± 0.08

a β refers to the overall reaction M + L + *i*H = MLH_*i*_ (charges omitted for simplicity); ±standard deviation.

It is clear that the stability of all the species slightly decreases by increasing the temperature. As an example, log β_113_ is equal to 30.02 and 29.76 at *T* = 288.15 and 318.15 K, respectively.

These results are further strengthened by spectrophotometric titrations in the range 210 < λ < 350 nm. By means of spectrophotometry, the speciation model has been verified by performing titrations on the ligand, together with specific amounts of NaCl and HCl, in order to establish the molar absorption coefficients of the LH, LH_2_, LH_3_, and LH_4_ species and later on the mixture metal–ligand under the same experimental conditions. The obtained results, in agreement with the potentiometric ones in terms of both speciation model and stability constant values, are listed in Table 4.

**Table 4:** Comparison between Potentiometric and Spectrophotometric Results in NaCl at *I* = 0.15 mol L^–1^ and *T* = 298.15 K

	log β^a^	
species	potentiometric results	spectrophotometric results
MLH_3_	29.97 ± 0.08	30.27 ± 0.04
MLH_2_	25.87 ± 0.09	26.32 ± 0.03
MLH	16.59 ± 0.09	17.16 ± 0.02

a β refers to the overall reaction M + L + *i*H = MLH_*i*_ (charges omitted for simplicity); ±standard deviation.

**Figure 3:**
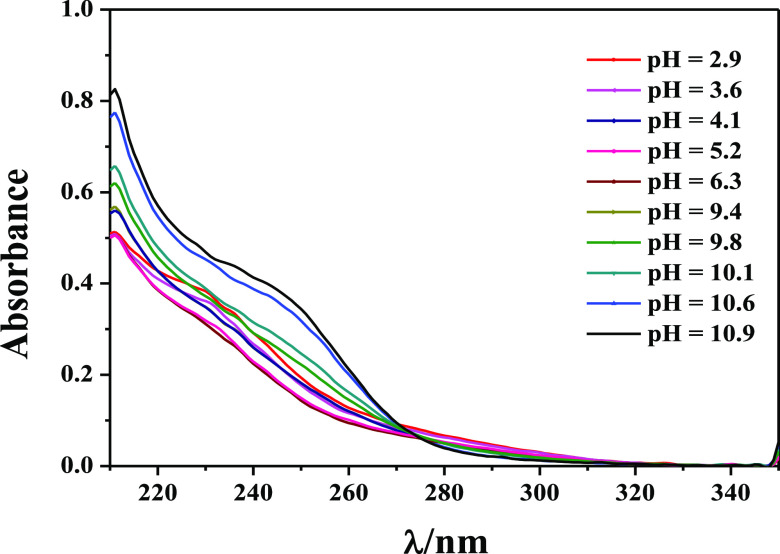
Example of spectrophotometric titration relative to As(III)-DMSA system. Conditions: *C*_M_ = 0.05 mmol L^–1^, *C*_L_ = 0.1 mmol L^–1^, *I* = 0.15 mol L^–1^ in NaCl, and *T* = 298.15 K.

**Figure 4:**
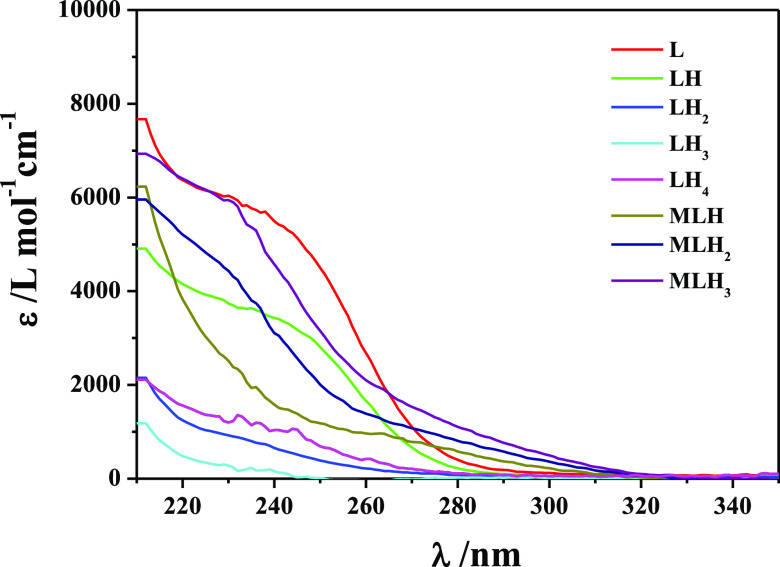
Molar absorption coefficients of As(III)-DMSA complexes together with those of the ligand species.

In Figure 3, an example of spectrophotometric curves at different pH values relative to the As(III)-DMSA system is shown. Molar absorption coefficients of H^+^ and As(III)-DMSA species are plotted in Figure 4.

Finally, in order to fully characterize this system from the thermodynamic point of view, all the thermodynamic parameters have been determined and are summarized in Table 5. As can be noticed, on the basis of the reaction 1, all the As(III) complexes exhibit negative enthalpies with values significantly higher than *T*Δ*S*, better pointed out in the histograms shown in Figure 6. Such an aspect highlights the presence of a strong interaction between DMSA and As(III), likely due to the two thiolic binding sites.

**Table 5:** Overall Thermodynamic Formation Parameters for H^+^- and As(III)-DMSA Species in NaCl at *I* = 0.15 mol L^–^^1^ and *T* = 298.15 K

reaction^a^	–Δ*G*^b^	Δ*H*^b^	*T*Δ*S*^b^
L + H = LH	62.8 ± 0.2	22 ± 3	84.8 ± 3
L + 2H = LH_2_	115.9 ± 0.2	40 ± 3	155.9 ± 3
L + 3H = LH_3_	136.2 ± 0.1	65 ± 2	201.2 ± 2
L + 4H = LH_4_	150.6 ± 0.2	57 ± 3	207.6 ± 3

M + L + 3H = MLH_3_	171.0 ± 0.5	–20 ± 3	151 ± 3
M + L + 2H = MLH_2_	147.6 ± 0.5	–32 ± 4	115.6 ± 4
M + L + H = MLH	94.9 ± 0.5	–50 ± 4	44.9 ± 4

a Charges omitted for simplicity.

b In kJ mol^–1^, ±standard deviation.

### Comparison with Monothiolic Ligands

The results obtained for DMSA are very different from those recently reported^13^ for monothiolic ligands (thiolactic acid, TLA, and thiomalic acid, TMA), for which the speciation models were featured by mono- and tri-coordinated species with lower stability constants. By considering the partial formation constants (log *K*_111_ = 5.58, log *K*_112_ = 5.55, log *K*_113_ = 6.10), the stability of the complexes considerably increases with respect to TLA and TMA ligands which present log *K*_111_ = 2.09 and 3.25, respectively.^13^

**Figure 5:**
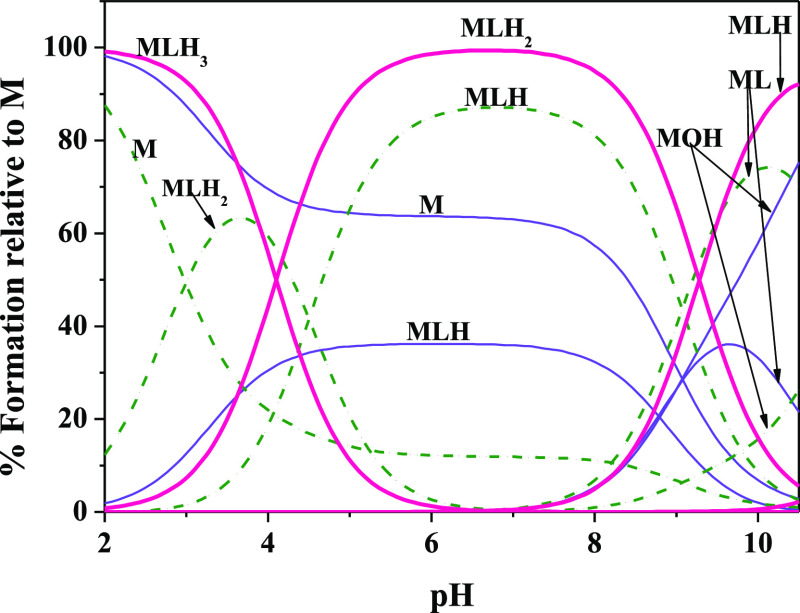
Species distribution relative to As(III)-TLA (thin violet line), -TMA (dashed green line), and -DMSA (thick pink line) systems. Conditions: *C*_M_ = 1 mmol L^–1^, *C*_L_ = 5 mmol L^–1^, *I* = 0.15 mol L^–1^ in NaCl, and *T* = 298.15 K.

The distribution diagram of the As(III)-DMSA species (Figure 5) further highlights the stability of the complexes, since the formation percentages of all the species (represented with a pink curve) reach values close to 100% and the di-protonated species (MLH_2_) results to be prevalent in all the investigated pH range. Certainly, the involvement of the second thiol group, as already reported in literature,^30^ is responsible for the greater stability that increases by increasing the number of carboxylic groups (one in TLA and two in TMA and DMSA) and especially by increasing the number of thiolic groups (one in TLA and TMA and two in DMSA).

**Figure 6:**
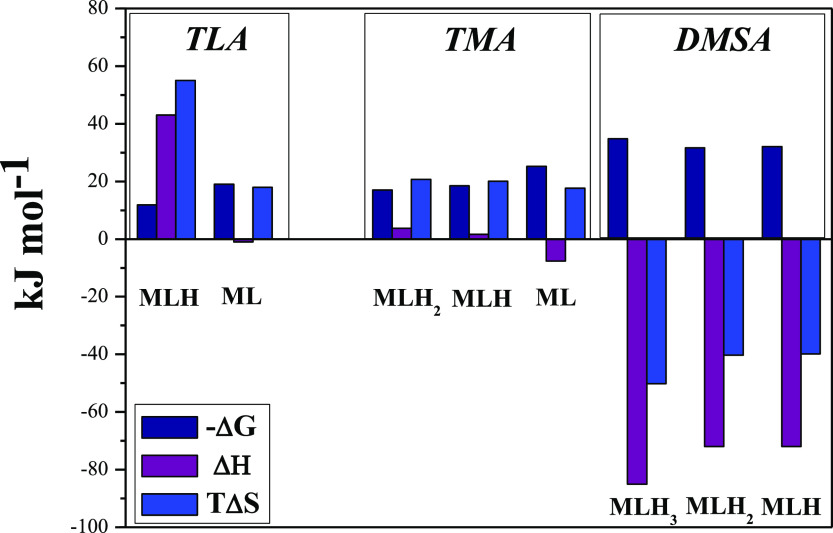
Thermodynamic parameters on the basis of the reaction 1, referred to the complexes As(III)-TLA (a), -TMA (b), and -DMSA (c), in NaCl at *I* = 0.15 mol L^–1^ and *T* = 298.15 K.

By comparing the partial thermodynamic parameters (Figure 6), all the formation processes of TLA and TMA complexes appear to be endothermic, with exception of ML, and the main contribution to the free energy is entropic since *T*Δ*S* > Δ*H*. This behavior suggests that the interactions are not pure soft–soft and that also the carboxylic group is involved in the chelation process.^13^ As for the As(III)-DMSA system, an opposite trend is observed since all the species exhibit negative enthalpies with values larger than *T*Δ*S*. As already mentioned, this aspect indicates the presence of a strong interaction between DMSA and As(III), likely due to the two thiolic binding sites. Moreover, negative entropy values could be ascribed to an increase of the order caused by the typical solvation process. Such an evidence has been further strengthened by the *ab initio* molecular dynamics simulations reported in the respective section.

### Sequestering Ability

Assessment of the speciation models makes it possible to quantitatively evaluate the ability of each ligand to sequester a given metal cation. In order to conduct such a kind of analysis in real systems—which are multicomponent solutions—the trivial comparison of the stability constants is clearly not sufficient mainly because of the presence of many other “interfering” ligands and cations that could lead to a series of competing reactions. For this reason, two metal–ligand systems featured by different formation constants, under certain conditions, may show the same formation percentages. Therefore, all the variables that may influence the formation of the complexes, such as the experimental conditions, the acid–base properties of the ligand and metal, competition with other metals and ligands which are simultaneously present in natural systems, have to be taken into account.

**Figure 7:**
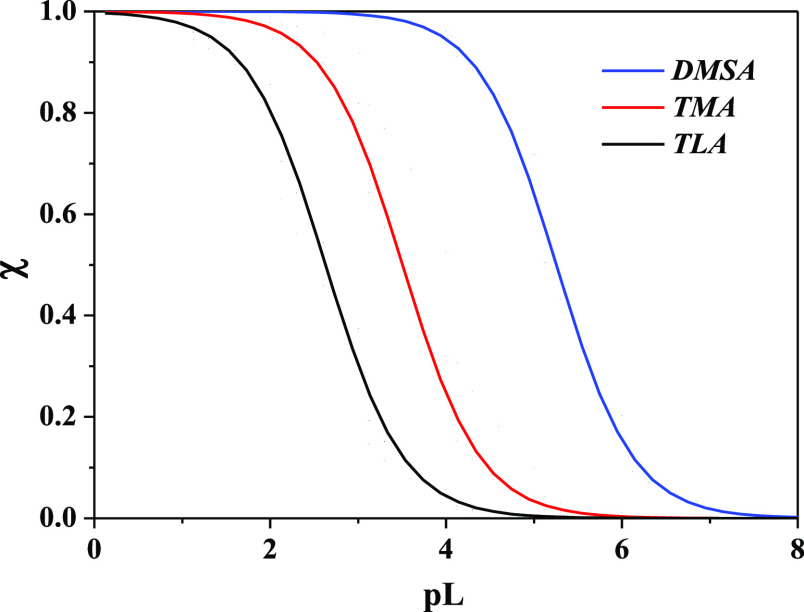
Sequestration diagram of As(III)-TLA, -TMA, and -DMSA species at *I* = 0.15 mol L^–1^ in NaCl, *T* = 310.15 K, and pH = 7.4.

In order to compare the sequestering ability of different ligands, the pL_0.5_ parameter can be used.^31,32^ pL_0.5_ is an empirical parameter that, once fixed the experimental conditions (ionic strength, ionic medium, temperature, pH, and metal concentration), provides an objective representation of the sequestering ability of a ligand toward a metal ion. It represents the total concentration (as antilogarithm) of the ligand necessary to “sequester” the 50% (in mole fraction, *x* = 0.5) of the metal cation concentration in trace, and it can be calculated by plotting the mole fraction (χ) of the metal complexed by the ligand as a function of pL (pL = −log [L], where [L] is total ligand concentration). This function is graphically represented by a sigmoid curve with asymptotes 1 for pL → −∞ and 0 for pL → +∞:
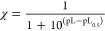
2Examples and applications of pL_0.5_ parameter can be found in refs (31, 33−40). Sequestering diagrams of mono- (TLA, TMA) and dithiolic (DMSA) ligands toward As(III)_,_ obtained by reporting the mole fraction of As(III) complexed by each ligand are shown in Figure 7. Diagrams are simulated at pH = 7.4, *I* = 0.15 mol L^–1^, and *T* = 310.15 K in order to evaluate the sequestering ability of ligands in conditions that simulate those of biological fluids. As evidenced for the complexing ability, also the sequestering ability toward the metal cation present in traces follows the trend TLA < TMA ≪ DMSA (with pL_0.5_ = 2.62, 3.50, and 5.25, respectively) and, therefore, it increases by increasing the number of carboxylic groups (i.e., from TLA to TMA) and of thiol groups (i.e., from TMA to DMSA). Results highlight that, from the thermodynamic point of view, DMSA is the best chelating agent among other −SH ligands.

### *Ab Initio* Molecular Dynamics Simulations

**Figure 8:**
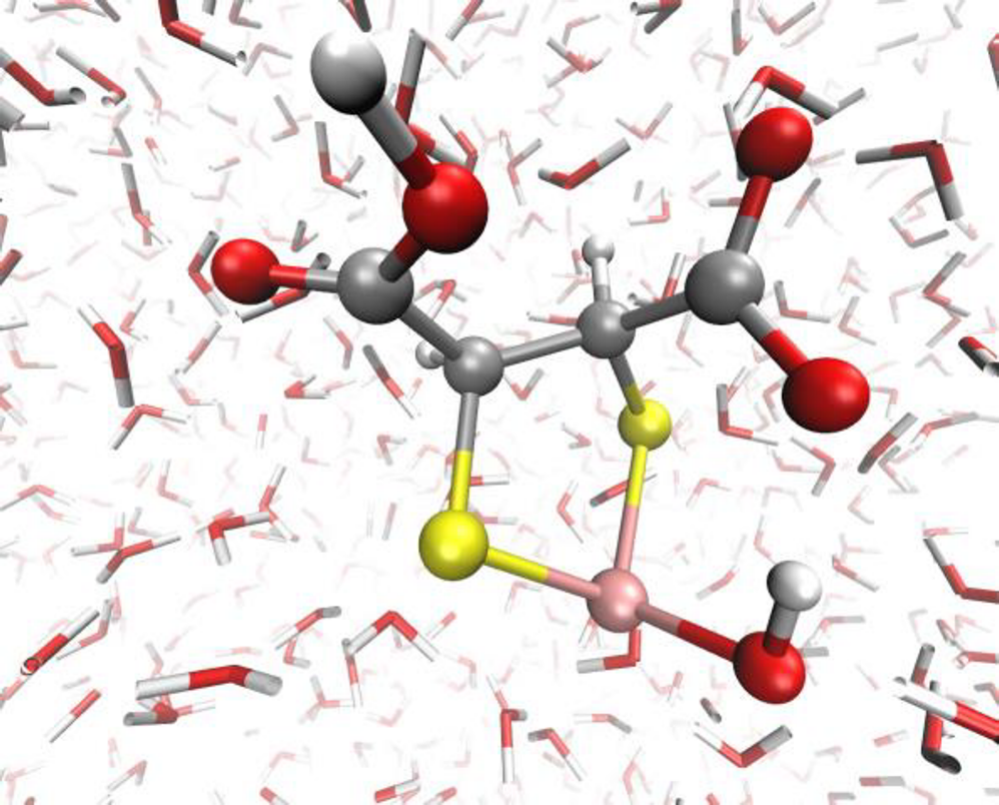
Typical As(III)-DMSA complex observed by means of *ab initio* molecular dynamics simulations. Red, white, pink, and yellow spheres represent oxygen, hydrogen, arsenic, and sulfur atoms, respectively.

In order to definitely clarify some crucial aspects emerging from the thermodynamic experiments presented in the previous section, prolonged *ab initio* molecular dynamics simulations were performed on the As(III) species chelated by DMSA species. Contrarily to the cases reported for TLA and TMA,^13^ the carboxylic groups do not *directly* come into play in the binding of arsenic, the latter being tightly chelated by *both* sulfur atoms, as shown in Figure 8.

From our first-principles molecular dynamics tests such a molecular configuration appears to be the most likely under the idealized neutral conditions reproduced in our simulations. However, one of the carboxylic groups holds a key place in the overall process of stabilization of the complex As(III)-DMSA. In fact, once the thioacid chelates the arsenic atom, one of the carboxylic groups suddenly deprotonates in favor of the solvent. As a direct consequence, once an hydroxide group binds As(III) (similarly to the previous cases for TLA and TMA^13^), the OH^–^ moiety rapidly establishes a strong H-bonded interaction with the deprotonated carboxylic group of DMSA, as shown in Figure 8. The latter molecular configuration was hold up to the end of the respective simulation (100 ps), suggesting that an improved stability of the complex was achieved under such a circumstance. Moreover, the fact that both thiolic and carboxylic groups are responsible for the coordination of As(III) with TLA and TMA species, while in the presence of the DMSA molecule both thiolic groups are involved, would explain the trend of the thermodynamic parameters presented in the previous section and the increase in terms of stability of the species by going from the mono- to the dicarboxylic ones (TLA vs TMA and DMSA) but especially from the mono- to the dithiol ligands (TLA and TMA vs DMSA).

**Figure 9:**
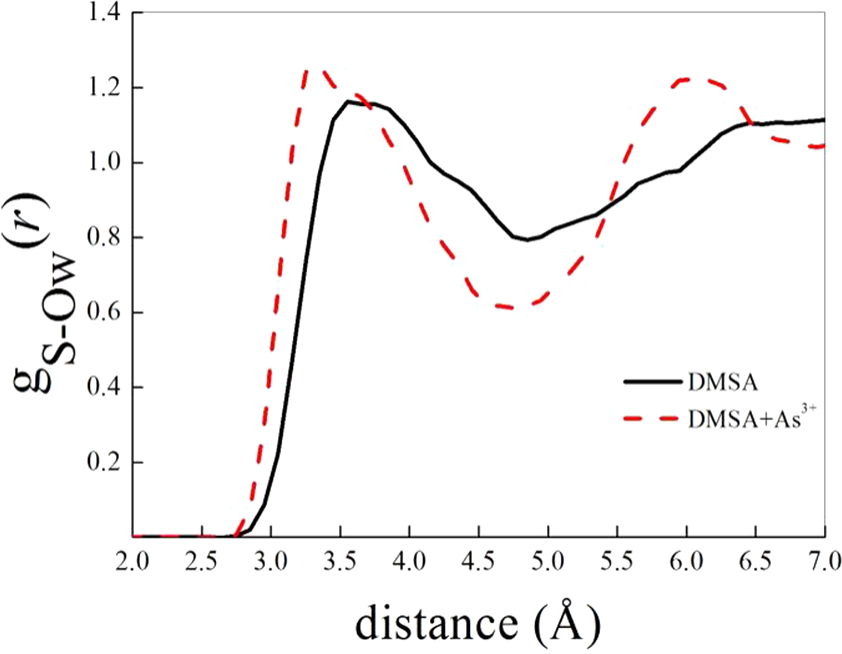
Atomistic (i.e., sulfur–water oxygen) radial distribution functions of pure DMSA in aqueous solution (black curve) and of an As(III)-DMSA complex (as that shown in Figure 8) in water (red dashed curve).

Finally, such a kind of investigation is able to shed light on another crucial evidence emerged from the presented experiments indicating that a negative entropy change could be a result of an order increase due to the solvation process. As plotted in Figure 9, the sulfur–water oxygen radial distribution function *g*_S-Ow_(*r*) clearly shows an evident more structured (i.e., more ordered) local water environment around DMSA species when the ligand chelates As(III). In fact, not only the locations of all the peaks and of all the dips of *g*_S-Ow_(*r*) shift to smaller distances, but also more pronounced saddle points characterize its profile. The latter finding hugely strengthens the indications stemming from the data emerged in the previously presented calorimetric and spectrophotometric experiments.

## Conclusions

The purpose of this paper is to provide an adequate thermodynamic analysis regarding the interaction of As(III) with DMSA, a molecule already employed for lead detoxification, in order to have a more complete framework for its possible usage as a detoxifying agent against As(III) in the human body. This knowledge of the thermodynamic behavior, together with the mechanism of the interaction, represents a starting point for further developments.

In particular, an in-depth comparison with monothiol ligands (TLA and TMA) was performed, underlining that the stability constants of all the species relative to the As(III)-DMSA system showed larger values than those reported for systems in which As(III) interacts with monothiols, thus strengthening the already known idea that As(III) exhibits a preference for dithiol ligands. This aspect was also confirmed by thermodynamic results which displayed a pronounced exothermic trend, in terms of enthalpy values, with respect to the monothiol systems, pointing out that a strong interaction occurs between As(III) and the ligand under study. A further confirmation was also given by *ab initio* molecular dynamics simulations which showed that As(III) interacts with both sulfur atoms while the carboxylic groups do not take directly part in the chelation process. Moreover, the analysis of the sulfur–water oxygen radial distribution function *g*_S-Ow_(*r*) clearly evidenced a more structured local water distribution around the complexes compared to the ligand alone that may explain the negative entropy values experimentally observed, generally attributed to an increase of the order caused by the solvation process. Finally, in order to better highlight the marked affinity of As(III) toward DMSA, the sequestering ability of the ligand toward the metalloid was calculated at physiological conditions, and the comparison with TLA and TMA showed, once again, the best performance of DMSA.
